# Appropriate antibiotic use and antimicrobial resistance: knowledge, attitudes and behaviour of medical students and their needs and preferences for learning

**DOI:** 10.1186/s13756-023-01251-x

**Published:** 2023-05-17

**Authors:** Miriam Wiese-Posselt, Thiên-Trí Lâm, Christin Schröder, Sandra Schneider, Oliver Kurzai, Markus A. Feufel, Petra Gastmeier

**Affiliations:** 1 Institute of Hygiene and Environmental Medicine, Charité – Universitätsmedizin Berlin Corporate Member of Freie Universität Berlin, Humboldt-Universität zu Berlin, Berlin, Germany; 2 Institute for Hygiene and Microbiology, University of Würzburg, Würzburg, Germany; 3 Division of Ergonomics, Department of Psychology and Ergonomics (IPA), Technische Universität Berlin, Berlin, Germany

**Keywords:** Appropriate antibiotic use, Medical students’ undergraduate education, Antimicrobial resistance

## Abstract

**Background:**

The impact of an appropriate use of antibiotics on the prevention of antimicrobial resistance (AMR) has been demonstrated. Surveys have shown, however, that medical students do not feel sufficiently trained to use antibiotics wisely. The aims of our study were (1) to describe what medical students currently know about appropriate antibiotic use, and (2) to identify students’ learning preferences as a basis for developing student-centred teaching modules to convey the basics of AMR prevention.

**Methods:**

We performed an online survey at Charité Universitätsmedizin Berlin and the Julius-Maximilians-University Würzburg on the knowledge, attitudes, and behaviour (KAB) of medical students concerning AMR, antibiotic treatment options, and their perceptions of AMR topics addressed in the medical curriculum. Participants were able to fill out an online questionnaire between December 2019 and February 2020. In addition, we conducted focus group discussions with lecturers and medical students in winter 2019/2020 to identify AMR-related learning needs and preferences. Data were analysed descriptively.

**Results:**

Overall, 356 students (response rate 5.1%) participated in the KAB survey. Of these, 192 (54%) strongly agreed that the topic of AMR is relevant to students’ clinical practice and 48% (171/355) stated that their future antibiotic prescription behaviour will have an influence on AMR development in their region. Participating students seemed to be interested in the topic of AMR and antibiotic therapy. But even of them, only 46% answered the question about the length of antibiotic use for community-acquired pneumonia correctly and 57% the question about the appropriate use of antibiotics in *Staphylococcus aureus* infections. Focus group discussions with students (n = 7) and lecturers (n = 9) identified a lack of competence in the responsible use of antibiotics and the prevention of AMR. Respondents stated that the teaching formats and AMR-related content should emphasize clinical applications, interaction with peers/clinicians, and repeated formative feedback from instructors.

**Conclusions:**

Our results show that even medical students who were interested in the AMR problem were not able to use antibiotics appropriately due to gaps in knowledge and a lack of clinical skills. Based on the insights gained in the learning preferences of students and their content priorities, improved student-centred teaching materials should be developed.

**Supplementary Information:**

The online version contains supplementary material available at 10.1186/s13756-023-01251-x.

## Background

Antimicrobial resistance (AMR) represents a global threat to human health. International health organisations are calling for measures to limit the further emergence of resistant microorganisms [1, 2]. Misuse or overuse of antibiotics is a crucial driver of AMR [3]. Therefore, one important measure to be taken is antibiotic stewardship in both the inpatient and outpatient sectors. This includes not only rules and processes for the wise handling of antibiotics but also targeted communication skills and distinct decision-making competence of individuals involved in patient care. Because today’s medical students are tomorrow’s practitioners and prescribers, it seems of great importance to train them in appropriate antibiotic use during their studies. In recent years, several surveys have reported on medical students’ knowledge, attitudes and behaviour regarding AMR and the appropriate use of antibiotics [4–8]. In addition, several relevant approaches and projects in medical undergraduate education were reported and discussed [9–13]. The surveys and studies showed that medical students, especially those in advanced semesters, have been able to acquire knowledge about antibiotics but lack both decision-making skills when it comes to negotiating antibiotic use with patients as well as practical competences in the inpatient treatment of patients. The research cited here aimed at describing the status quo in knowledge, attitudes and behaviour (KAB) among medical students as well as their expectations when taught about wise antibiotic therapy. Other studies have explicitly tested individual learning tools. We, in contrast, wanted to build on the status quo assessment of KAB in a specific student population by finding out how and with what formats students would like to learn about antibiotic therapy and AMR. Therefore, the aims of our study were (1) to find out what knowledge medical students have about AMR and appropriate antibiotic use, as well as what competencies and skills they are taught for adequate antibiotic treatment; and (2) to identify students’ preferences for teaching formats and AMR-related learning needs. To analyse students’ KAB, we conducted an online survey among medical students from two major medical universities in Germany (Charité—Universitätsmedizin Berlin (CUB) and the Julius-Maximilians-University Würzburg (JMU)). In addition, focus group discussions with students and lecturers were performed to identify AMR-related teaching requirements and the learning formats students prefer. The results are reported here with an emphasis on the teaching contents and formats that are needed to increase medical students’ knowledge and skills regarding appropriate antibiotic use. These activities were performed as part of the network project RAI (Responsible antibiotic use via information and communication), InfectControl, which has been described in detail elsewhere [14, 15].

## Methods

### Survey of knowledge, attitudes, and behaviour (KAB survey)

A questionnaire for the KAB survey was developed for medical students based on the results of a literature review and of surveys that had already been administered to physicians and general practitioners (GP) in the RAI project [14, 16, 17]. Scientists and experts in medical education at CUB and JMU designed and finalized the questionnaire after an extensive review process. The questionnaire contained 22 thematic questions. In addition, demographic data about the participating students was collected. All questions about student attitudes and behaviour were formulated using Likert scales. The questionnaire focused on the following AMR and antibiotic use topics: (a) problem perception, (b) barriers to implementation, (c) knowledge, and (d) information retrieval/knowledge acquisition. To assess students’ problem perception, we included six items about their personal awareness of AMR as well as their behaviour in connection with this problem. We also asked whether the students have already had contact with patients with AMR. In the questionnaire section on barriers to appropriate antibiotic use, we asked in which areas (e.g., human or veterinary medicine) action should be taken to curb antibiotic use or misuse. We inquired whether students have already raised the issue with patients with infectious diseases with whom they had contact as part of their studies or internships and whether the topic had been raised during their own last visit to the doctor for an infection. We assessed students’ knowledge about antibiotic therapy for common infectious diseases using four multiple-choice questions. In the questionnaire section on information retrieval/knowledge acquisition, we collected data on the sources students use for information on antibiotic therapy and AMR, and whether the students had been able to acquire sufficient knowledge on these topics thus far during their studies. Moreover, we asked the students which teaching formats they considered suitable and what content should be provided on AMR and antibiotic treatment.

Using the open-source software LimeSurvey, the online questionnaire was made available to all medical students enrolled at the time at CUB (n = 4797) and JMU (n = 2236). The survey was advertised via student council mailing lists with a link to the anonymous online survey. It was sent out in December 2019, again in January 2020 (as a reminder), and was closed at the end of February 2020. Answers to all questions were analysed with descriptive statistical methods (percentage, absolute counts). Data were aggregated over all answers and grouped by university (i.e., CUB, JMU) and study level (i.e., early semesters 1–5 vs. advanced semester 6 or higher). To investigate differences between early and advanced semesters, a Fisher’s exact test was conducted. Analyses were performed using R [R foundation, Vienna, Austria].

### Focus group discussions

The focus group discussions with lecturers and medical students were organized in cooperation with an agency that specializes in qualitative market research (Point blank Research and Consulting Agency, Berlin). Prior to the discussion sessions, the agency staff conducted individual interviews with young physicians who worked as doctors during their first or second year after graduation. The subsequent focus group discussions with lecturers took place in November 2019. Participants were faculty members from the departments of internal medicine, infectious disease, microbiology, infection prevention and control, and pharmacy. The institutions they represented were CUB, the JMU, the Free University of Berlin (Institute of Pharmacy), and the Vivantes Hospital Group Berlin. In January 2020, a focus group discussion was conducted with seven medical students from both early and advanced semesters of CUB. Both focus group discussions were conducted by a staff member of the Point blank Research agency to avoid researcher bias. Scientists from CUB and Technische Universität Berlin observed the discussion sessions through a one-way mirror window. The agency subsequently provided a descriptive summary of the main points of the focus group discussions, which form the bases for the results reported below.

## Results

### KAB survey

In total, 356 students participated in the KAB survey (overall response rate of 5.1%), 273 were students at JMU (response rate of 12.2%) and 83 at CUB (response rate of 1.7%). Details on demographics are shown in Table 1. The sample was split into students in early semesters (semester 1–5) and advanced semesters (6+). Here, we present the overall results of the KAB survey as well as those for the subgroups of early and advanced semesters. All results, including those showing the differences between the two universities CUB and JMU, are reported in the Additional file 1.

**Table 1:** Study participants of the KAB survey, Germany 2019–2020

	Total	CUB	JMU	Early semesters^a^	Advanced semesters^a^
	356	83	273	128	228
CUB				46	37
JMU				82	191
Semester					
Median [IQR]	7 [5, 10]	5 [3, 8]	8 [5, 10]	3 [2, 5]	9 [7, 11]
Gender					
Diverse (%)	3 (0.8)	1 (1.2)	2 (0.7)	0	3
Male (%)	106 (29.8)	19 (22.9)	87 (31.9)	33	73
Female (%)	244 (68.5)	62 (74.7)	182 (66.7)	93	151
Not specified (%)	3 (0.8)	1 (1.2)	2 (0.7)	2	1
Work experience^b^					
Yes (%)	99 (27.8)	17 (20.5)	82 (30.0)	37	62
No (%)	248 (69.7)	65 (78.3)	183 (67.0)	88	160
Not specified (%)	9 (2.5)	1 (1.2)	8 (2.9)	3	6

*CUB* Charité—Universitätsmedizin Berlin (CUB), *IQR* Interquartile range, *JMU* Julius-Maximilians-University Würzburg

^a^Early semesters were defined as semesters 1–5, advanced semesters as semester 6 onwards

^b^Work experience in the healthcare field such as nursing, physiotherapy or emergency medicine

**Fig. 1:**
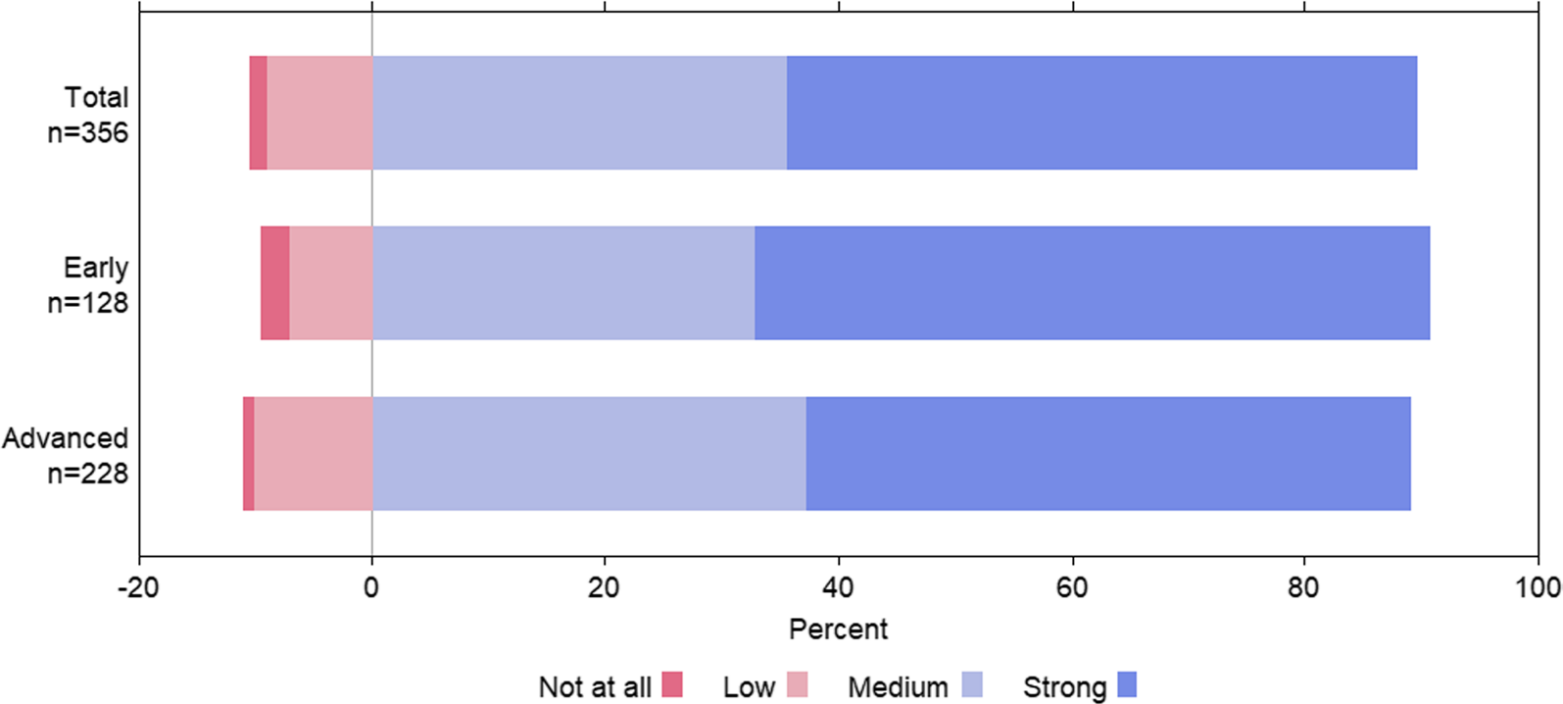
Students' assessment of the level of the relevance of antibiotic resistance in the context of their studies and/or clinical internships. KAB survey, Germany 2019–2020. Early = semesters 1–5, Advanced = semesters from semester 6 onwards. No significant difference between students in early and advanced semesters (Fisher’s Exact Test, *p* = 0.3815)

**Fig. 2:**
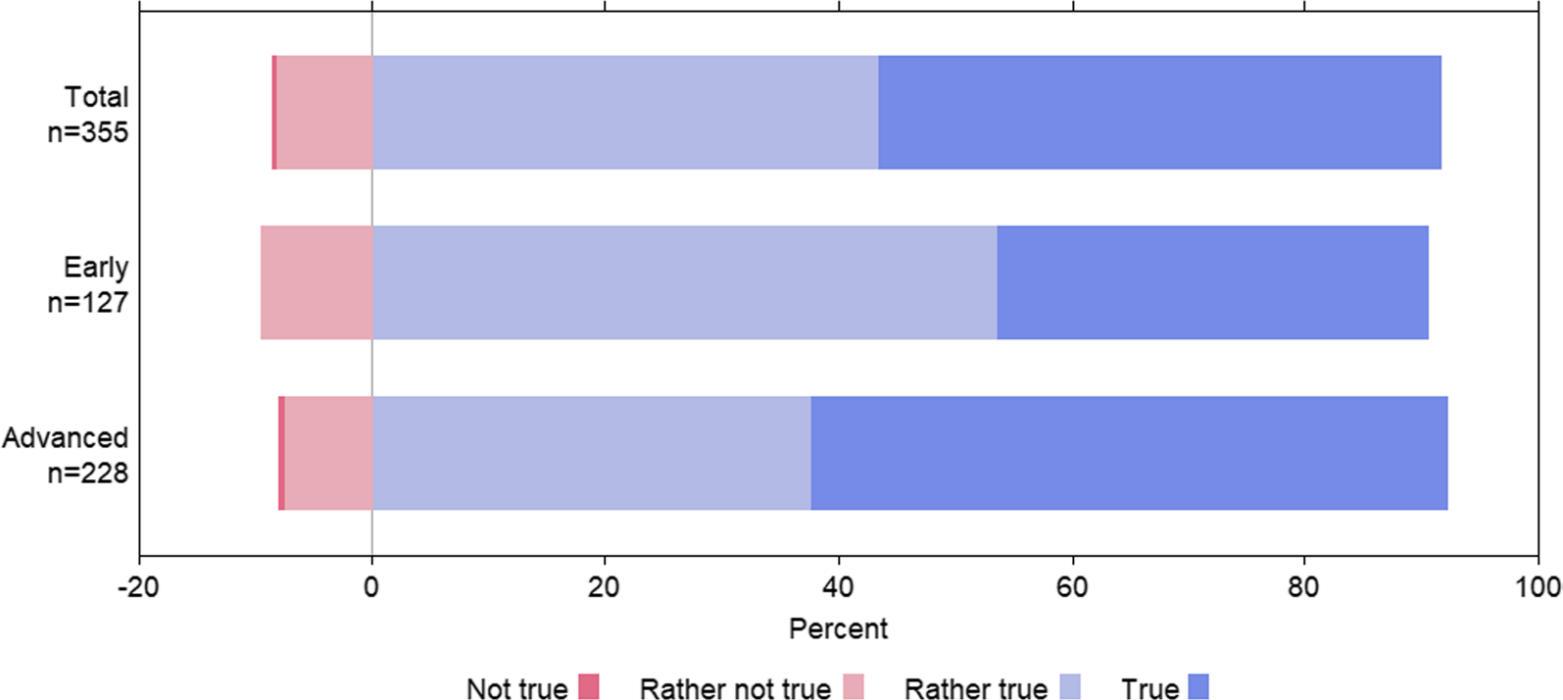
Medical students’ opinion on whether their future antibiotic prescribing behaviour will have an influence on the antibiotic resistance situation in their region. KAB survey, Germany 2019–2020. Early = semesters 1–5, Advanced = semesters from semester 6 onwards

In the questionnaire section on problem perception it became apparent that the majority of the participating students considered AMR an emerging health challenge and were aware that appropriate antibiotic use is an important means of addressing it (Figs. 1 and 2).

As shown in Fig. 2, students from the early semesters were significantly less likely than students from advanced semesters to define AMR as a factor that would influence their future prescription behaviour (Fisher’s Exact Test *p* = 0.0075).

**Fig. 3:**
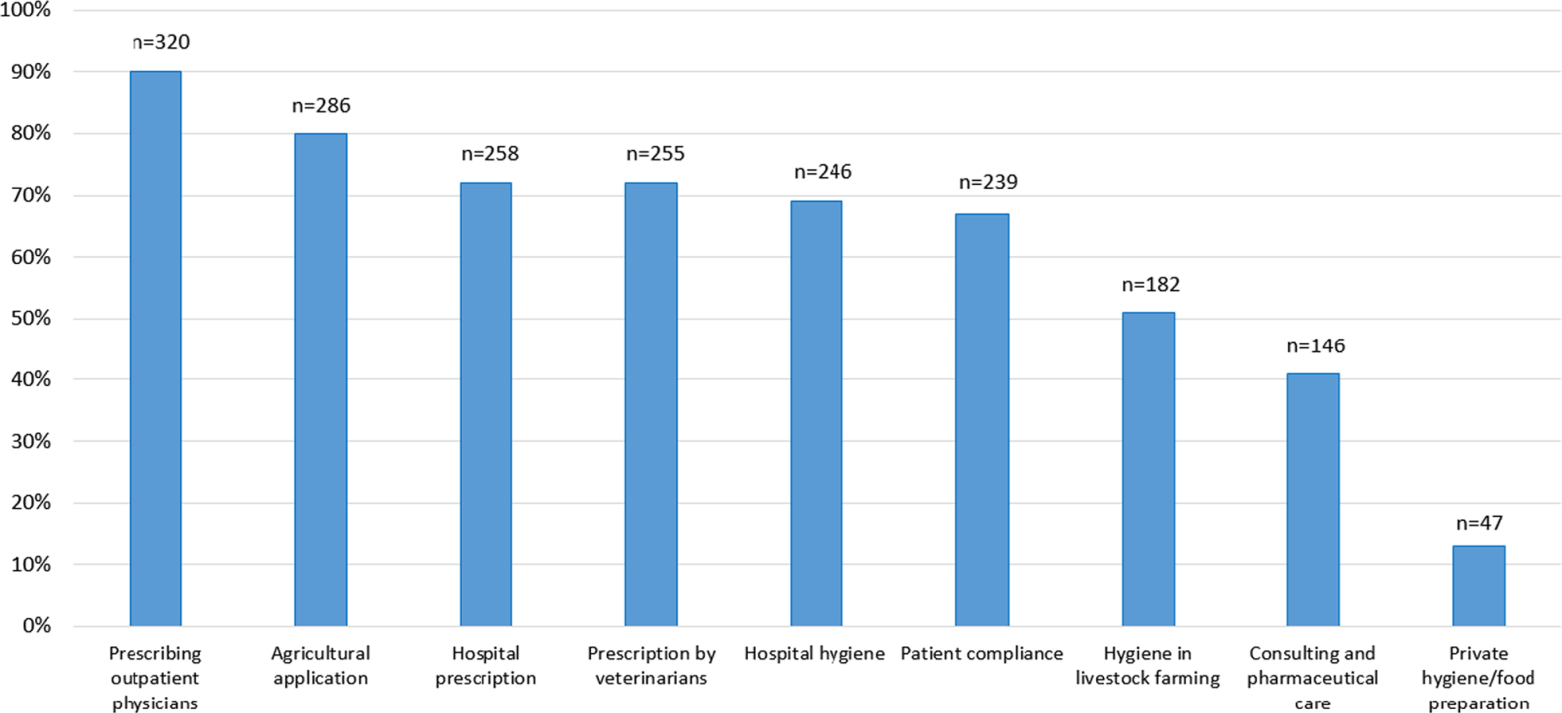
Areas where action should be taken to curb the increase in antimicrobial resistance: Answers of n = 356 students, multiple answers possible. KAB survey, Germany 2019–2020

In the questionnaire section on barriers to the implementation of appropriate antibiotic use and the reduction of AMR, we asked the students to choose the areas in which action should be taken to curb the increase of AMR. The majority of students recognized that action should be taken in almost all areas mentioned (Fig. 3).

Overall, 106 of the 356 students had consulted their GP for an infectious disease during the previous year. Of those 106 students, 59 (56%) were prescribed an antibiotic, 54 (92%) of whom took the medication as instructed by their physicians. Of the 356 students, 36 (10%) had taken an antibiotic without a doctor's prescription. They procured the antibiotic on their own, bought it abroad without a prescription, or used leftover drugs.

The responses to the knowledge questions showed the gaps in knowledge of both early and advanced semester students in the adequate antibiotic therapy of common infectious diseases (see Table 2). The differences in the rate of correct answers between students of early semesters (1–5) and advanced semester (6+) were significant for all questions (*p* < 0.0001).

**Table 2:** Knowledge on therapy/practice in the use of antibiotics. Overview of the rate of correct answers to the knowledge questions in total and broken down into early and advanced semester students

	Correct answer of the students			*p*-value (early and advanced semesters)
	Total n = 356	Early semesters (1–5), n = 128	Advanced semesters (6+), n = 228	
*Question*				
The following antibiotic is considered the antibiotic of first choice for infections with *Staphylococcus aureus*;—correct answer: Flucloxacillin	203 (57%)	32 (25%)	171 (75%)	< 0.0001
Acute bronchitis that persists for more than one week is usually an indication of antibiotic therapy.—Not true!	209 (59%)	41 (32%)	168 (74%)	< 0.0001
Acute pyelonephritis in a young patient without concomitant diseases is a clear indication for antibiotic therapy.—True!	201 (56%)	39 (31%)	162 (71%)	< 0.0001
Antibiotic therapy longer than 7 days is usually not necessary in the treatment of community-acquired pneumonia when there is clinical response.—True!^a^	164 (46%)	28 (22%)	136 (60%)	< 0.0001

KAB survey, Germany 2019–2020

^a^At the time of the survey, the national guidelines for the treatment of community-acquired pneumonia (CAP) in adults generally recommended a treatment duration of 7 days in the case of clinical response to the antibiotic therapy. Since 2021, there has been an updated guideline that recommends a treatment duration of 5 days for CAP if a response to the antibiotic therapy has already started [18]

**Fig. 4:**
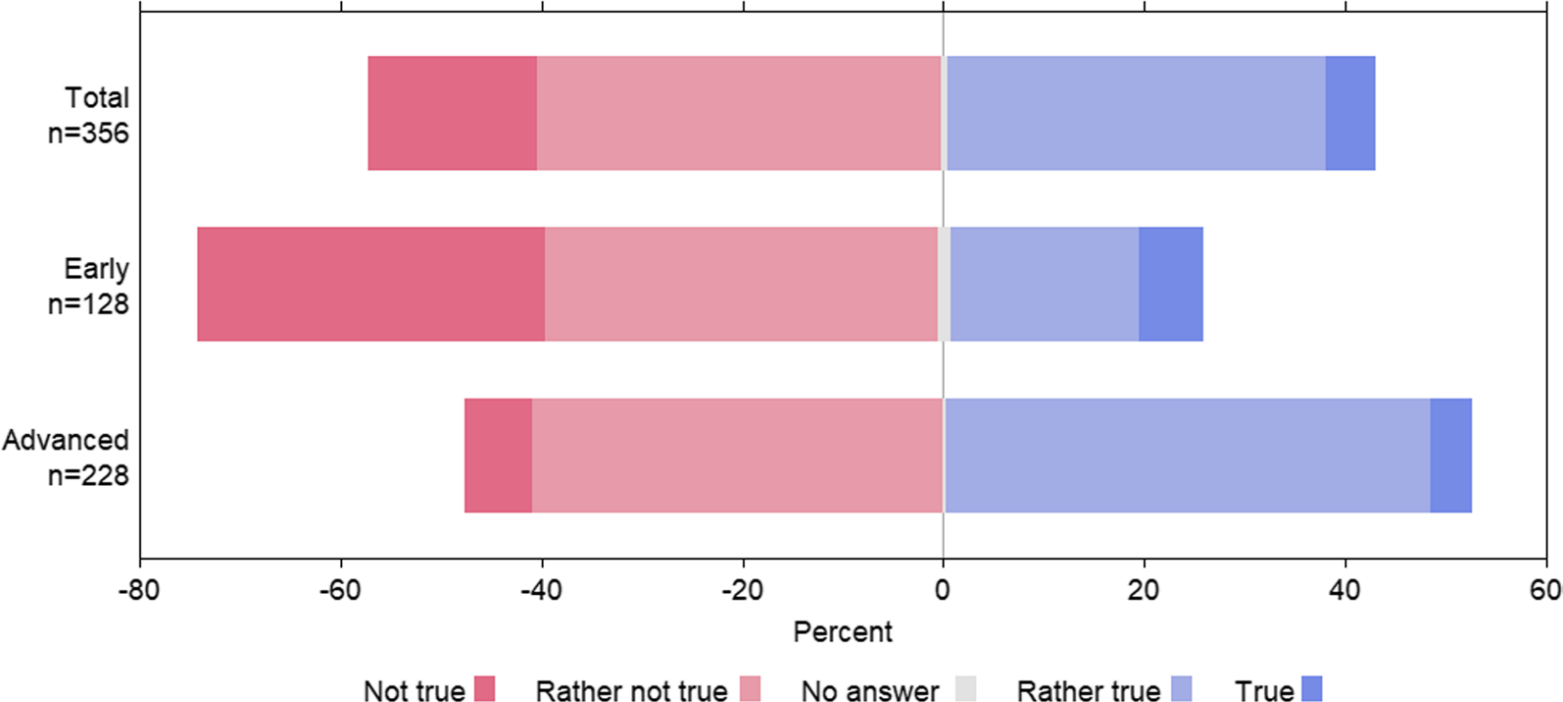
Assessment of the students to what extent the previous knowledge acquisition on the topic of appropriate antibiotic use and antimicrobial resistance is sufficient. KAB survey, Germany 2019–2020. Early = semesters 1–5, Advanced = semesters from semester 6 onwards

The results of the questionnaire section on information source/knowledge acquisition showed that only 5% of all participating students stated that they had acquired sufficient knowledge of antibiotic therapy and AMR thus far. There was a significant difference between the responses of students in early and advanced semesters (Fisher’s Exact Test, *p* < 0.0001), see Fig. 4.

Responses to the questions about information sources and media on AMR included classic sources and materials such as a learning software for preparing for the final medical examination in Germany (AMBOSS). In the case of electronic media, classic websites and search engines were favoured (321/356). With regard to medical education at university, the students also showed a high affinity to classic teaching formats such as lectures or seminars (Table 3). The answers to these three items did not vary between students of early and advanced semesters.

**Table 3:** Learning sources and formats for appropriate antibiotic use and AMR

**What sources do you use to learn about antibiotic therapy and the development of antimicrobial resistance?**								
*AMBOSS*-Online tool	Univer-sity course	Exchange with fellow students	Medical textbooks	Medical guide-lines	Subject- specific websites	Websites for laymen	No time	No need
266 (75%)	232 (65%)	175 (49%)	174 (49%)	152 (43%)	98 (27%)	44 (12%)	49 (14%)	7 (2%)

**What media formats do you use for your studies in courses at the university or for your own studies at home?**				
Classic websites	Apps	Visual online tutorials	Podcasts	Blogs
321 (90%)	174 (49%)	121 (34%)	39 (11%)	12 (3%)

**I consider the following formats to be suitable for imparting knowledge during studies on the topics of appropriate antibiotic therapy and antimicrobial resistance (please select a maximum of 5 formats):**								
Seminar	Lecture at univer-sity	Training games	Learning-apps	E-learning	Bedside teaching	Specific websites	MOOC	Podcasts
300 (84%)	209 (59%)	167 (47%)	145 (41%)	145 (41%)	140 (39%)	121 (34%)	107 (30%)	67 (19%)

KAB survey. Germany 2019-2020. Answers of n = 356 students, multiple answers possible

Table 4 shows students’ opinions on what a course (e.g. a massive open online course (MOOC)) on appropriate antibiotic use and AMR should include.

**Table 4:** Favoured content of a course on appropriate antibiotic use and the development of antimicrobial resistance

Content of a course on appropriate antibiotic therapy and antimicrobial resistance	Number of students (total *n* = 356)
Guideline-based therapy	308 (86%)
Modes of action of antibiotics	296 (83%)
Principles of appropriate antibiotic therapy	285 (80%)
Features of individual antibiotics	247 (69%)
Interactive case studies	239 (67%)
Epidemiology of multidrug-resistant pathogens	182 (51%)
Resistance mechanisms	166 (47%)
Microbiological diagnostics	84 (24%)
Pharmacokinetics details	69 (19%)
Selection and transmission	50 (14%)

Multiple answers possible, n = 356 students. KAB survey. Germany 2019–2020

### Interviews and Focus group discussions

In terms of the teaching and learning contents necessary for appropriate antibiotic use, the results of the interviews and the focus group discussions can be divided into four major topics: Awareness of the problems caused by AMR, medical students’ knowledge of AMR and antibiotic therapy, students’ practical competence/skills, and their preferred learning formats.

### Awareness

Students stated—and the lecturers confirmed this assessment—that their medical training sensitised them to the problem of AMR as a global public health threat and the importance of appropriate antibiotic use. However, concrete strategies for avoiding resistance seemed not to be fully covered, either in the curriculum or in clinical training routines. Medical students reported feeling little personal responsibility or involvement in the issue of AMR, a result of the fact that medical training is always oriented toward the treatment of the individual patient and very rarely on systemic challenges like AMR. It seems that in medical training, appropriate antibiotic use plays only a marginal role compared to the many other clinical competencies that are needed on a day-to-day basis. Also, most students reported their studies were focussed on the next exam rather than on issues important for their professional future. As a result, when they start their careers in the clinic, there is great uncertainty among medical graduates about appropriate antibiotic therapies that will prevent AMR.

### Knowledge

In addition to the basics of bacteriology and microbiological diagnostics, two areas of knowledge necessary for appropriate antibiotic use were identified by the students, lecturers, and young physicians. One was knowledge of antibiotics, e.g. their classification, mechanisms, and spectrum of activity. Another area reported in the focus group discussions was clinically-relevant knowledge of the pharmacokinetics and pharmacodynamics of antibiotics—it was noted that the dosage as well as possible interactions and side effects are important for the appropriate use of antibiotics.

### Competences

Students reported that because decisions about antibiotic therapy have to be made quickly in their daily work, there is often little time to reflect on those decisions and to seek advice from superiors. Therefore, from their point of view, medical competences and problem-solving skills must be trained. Medical competences start with the ability to take a patient’s medical history appropriately. This is the basis of differential diagnosis, which precedes empirical antibiotic therapy. The problem-solving skills relate to deciding for or against antibiotic therapy, to selecting the appropriate drug, and to the right timing for the start of antibiotic therapy. The students reported that a ‘fear of doing nothing’ is a major obstacle to implementing a more prudent use of antibiotics. Moreover, problem-solving skills include seeking information in the event of uncertainty, insecurity, or a lack of knowledge. It is important to know the right sources of information (e.g. guidelines) and the experts to consult (e.g., colleagues, supervisors). Patient management skills are also important—both to counter a patient’s desire to receive antibiotics when they are not needed and to convince a patient to take necessary antibiotics when he or she does not want therapy.

### Learning formats

The results of the discussions on suitable teaching formats and learning material revealed the following four main characteristics that should be considered for an effective AMR training program:

- *Practical relevance, focus of application* The direct transferability of what is learned to everyday clinical practice was rated as extremely important if learning formats and teaching content are to have their full effect.
- *Face-to-face instruction and interaction, peer group learning* Students prefer learning “offline,” face to face, together with experts and in interaction with real patients. Formats such as simulation training or bedside teaching were emphasized as particularly instructive. Peer-teaching formats in which students work with other students were also appreciated.
- *Feedback mechanisms and performance monitoring/learning control* Regular feedback on one’s own learning progress was considered particularly important and motivating. It is important that the feedback is not merely for the sake of feedback or performance evaluation (summative feedback), but that it reliably indicates whether competences relevant to future professional life have been acquired (formative feedback).
- *Little time outside the university curriculum* Because medical students have little free time, the willingness to study additional topics outside the curriculum, for example in a course at summer school, is low. If an issue is important, it should thus be part of the university curriculum.

## Discussion

Several reviews have summarised the results of KAB surveys of medical students thus far. We found that students who decided to participate in our survey understood the importance of AMR but lacked the knowledge and confidence to perform adequate antibiotic therapy [19–21]. Moreover, we can report that these students were aware of the importance of preventing of AMR (Fig. 1) and, therefore, indicated that the areas of human and veterinarian medicine, agriculture, livestock farming, and private/food preparation hygiene should be addressed in medical education and training to curb the increase in AMR (Fig. 3). A KAB survey of 357 medical students from 28 European countries showed even less awareness of the influence of veterinary medicine and livestock on the AMR situation than the students in our survey [22]. However, in the fight of AMR it is crucial that students understand and internalise the connection between the different sectors (humans, animals, plants, and environment) [23]. The approach toward maintaining the health of all sectors is called the “One Health” concept. Over 80% of respondents in the European survey stated they had never heard of it [22].

The observation that more than 90% of the students who participated in our KAB survey felt that their future antibiotic prescription behaviour will definitely or probably have an impact on AMR development in their region shows their awareness of the AMR problem (Fig. 2). However, the students do not feel sufficiently prepared by their medical studies to use antibiotics responsibly and thus to make an important contribution to the prevention of AMR. In our KAB survey, we specifically determined gaps in knowledge regarding questions about antibiotic therapy for common infectious diseases (Table 2). Overall, less than 60% of the respondents were able to answer the knowledge questions correctly; even among students in the advanced semesters, only between 60 and 75% knew the correct answers. This observation has also been made in other studies [21, 22, 24]. For example, it is reported in a survey of final year students at European universities that 47% of students would have prescribed inappropriate antibiotic therapy for patients with acute bronchitis and community-acquired pneumonia [24]. In our survey, the majority of students acknowledged that they had not acquired sufficient knowledge of appropriate antibiotic use and AMR in their previous studies (Fig. 4). The focus group discussions identified areas of knowledge and important competencies that should be taught in a focused manner to remedy these gaps in medical curricula. These include knowledge about the pharmacological principles of antibiotics, their modes of action, and their effect spectra, combined with the competencies and skills needed to apply this knowledge in clinical practice. In particular, students believed that practical and clinical competences and problem-solving skills should be taught more intensively. Competencies are defined as “trainable attributes of an individual that must be developed in order to successfully perform professional duties” [25]. Therefore, to implement more appropriate antibiotic use, it is crucial to develop teaching that is tailored to the needs of students and training formats to convey the skills and knowledge we identified as missing in medical curricula.

### Teaching formats and content

In our survey and focus group discussions, all conducted before the SARS-Coronavirus (CoV) 2 pandemic, students expressed a preference for analog teaching formats in small groups with a focus on practical skills, for example, bedside teaching. During the SARS-CoV 2 pandemic, virtual teaching formats have become increasingly important. The students provided detailed information about the virtual media they use for their education (Table 3). Different teaching formats seem to be considered suitable, depending on whether the student is acquiring new knowledge, memorizing facts, or learning practical skills and competencies. The following points were made by students as particularly relevant for imparting knowledge and skills in the field of appropriate antibiotic therapy and AMR:

- Clinical relevance of all teaching content
- Interaction with peers, possibility to question experts
- Regular formative feedback about practical knowledge and skills
- AMR and appropriate antibiotic use should not be merely supplemental but a regular part of the medical curriculum

In addition, the students indicated the content they felt should be offered in a course on appropriate antibiotic use and AMR (Table 4). Based on this information on effective teaching formats and content priorities, we plan to design undergraduate medical training modules that are tailored to students’ needs and preferences in AMR and antibiotic therapy. It will be important to evaluate these courses thoroughly in order to further improve and develop the AMR-related training of future health professionals.

## Limitations

For the KAB survey, the overall response rate was low at 5.1%. It is described in the literature that a low response rate in online surveys is an increasing phenomenon [26]. This is certainly due to the fact that in the field of teaching and research, online surveys are increasingly being sent to students, academics and others via email. Due to this low response rate, we must assume a selection bias. It is likely that mainly students who were interested in the topic of appropriate antibiotic use participated in our KAB survey and this aspect has biased the results of the knowledge questions. Although we are unable to assess the influence of selection bias on questions about attitude, behaviour and appropriate learning formats, the low level of knowledge among students who were aware of AMR and showed interest in the topic is even more alarming and supports our call for more targeted teaching materials and formats. Thus, we cannot and do not aim to generalize the results of our sample to other student populations. Instead, we suggest that despite the biases in the data, the KAB survey provides important insights into learning and teaching gaps among medical students that must be addressed to remedy the AMR challenge.

## Conclusions

The results of our KAB survey and focus group discussions indicate that even medical students who showed an awareness of the global AMR challenge lacked the knowledge and skills needed to use antibiotics wisely if AMR is to be mitigated. Based on these insights, we were able to identify teaching content and appropriate learning formats for training medical students how to use antibiotics wisely and, ultimately, help to prevent AMR.

## Supplementary Information

**Additional file 1**. Total data of the KAB survey.

## Data Availability

The data supporting the conclusions of this article is included within the article and its additional file. The report on the focus groups discussions is available from the corresponding author upon reasonable request.

## Abbreviations

AMR: Antimicrobial resistance
CAP: Community acquired pneumonia
CUB: Charité—Universitätsmedizin Berlin
GP: General practitioner
IQR: Interquartile range
JMU: Julius-Maximilians-University Würzburg
KAB: Knowledge, attitudes, and behaviours
RAI: Responsible antibiotic use via information and communication
SARS-CoV 2: SARS-Coronavirus 2
