# Supplementary material for: Appropriate antibiotic use and antimicrobial resistance: knowledge, attitudes and behaviour of medical students and their needs and preferences for learning
Source: Antimicrob Resist Infect Control. 2023 May 17;12:48. doi: 10.1186/s13756-023-01251-x (PMC10189209; doi:10.1186/s13756-023-01251-x)
Supplement: Supplementary file 1 — Additional file 1. Total data of the KAB survey. [file 13756_2023_1251_MOESM1_ESM.docx]

**Additional File 1**

**Results of the KAB survey, Germany 2019-2020**

1. **Topic 1: Antibiotic resistance and multidrug-resistant pathogens**

# 1.1 Who or what can become resistant to an antibiotic?

|  | Bacteria | Viruses | Bacteria and viruses |
| --- | --- | --- | --- |
| Total | 326 (92%) | 3 (1%) | 27 (8%) |
| Charité, Berlin | 80 (96% [90-100]) | 1 (1% [0-7]) | 2 (2% [0-8]) |
| Uni. Würzburg | 246 (90% [86-84]) | 2 (1% [0-3]) | 25 (9% [6-13]) |
| Early semesters | 122 (95% [90-98]) | 2 (2% [0-6]) | 4 (3% [1-8]) |
| Advanced semesters | 204 (89% [85-93]) | 1 (0% [0-2]) | 23 (10% [7-15]) |

# 1.2 How relevant is the topic of antibiotic resistance for you in the context of your studies and/or clinical internships?

|  | Not at all | Low | Medium | Strong |
| --- | --- | --- | --- | --- |
| Total | 5 (1%) | 32 (9%) | 127 (36%) | 192 (54%) |
| Charité, Berlin | 2 (2%) | 5 (6%) | 21 (25%) | 55 (66%) |
| Uni. Würzburg | 3 (1%) | 27 (10%) | 106 (39%) | 137 (59%) |
| Early semesters | 3 (2%) | 9 (7%) | 42 (33%) | 74 (58%) |
| Advanced semesters | 2 (1%) | 23 (10%) | 85 (37%) | 118 (52%) |

Charité, Berlin / Uni. Würzburg: Fisher's exact test 0.0320

Advanced semester / early semester: Fisher's exact test 0.3815

# 1.3 How often do you have contact with patients with multidrug-resistant pathogens during your studies at university?

|  | Monthly | Never | Rarely | Daily | Weekly |
| --- | --- | --- | --- | --- | --- |
| Total | 95 | 32 | 167 | 10 | 51 |
| Charité, Berlin | 22 | 10 | 45 | 1 | 5 |
| Uni. Würzburg | 73 | 22 | 122 | 9 | 46 |
| Early semesters | 26 | 21 | 72 | 2 | 6 |
| Advanced semesters | 69 | 11 | 95 | 8 | 45 |

Charité, Berlin / Uni. Würzburg: Fisher's exact test 0.0589

Advanced semester / early semester: Fisher's exact test <0.0001

# 1.4 Do you personally know people who have problems with multidrug-resistant pathogens?

|  | One person | Nobody | I do not know | Two or more persons |
| --- | --- | --- | --- | --- |
| Total | 61 (17%) | 224 (63%) | 24 (7%) | 47 (13%) |
| Charité, Berlin | 16 (19%) | 55 (66%) | 7 (8%) | 5 (6%) |
| Uni. Würzburg | 45 (16%) | 169 (62%) | 17 (6%) | 42 (15%) |
| Early semesters | 23 (18%) | 68 (53%) | 8 (6%) | 29 (23%) |
| Advanced semesters | 38 (17%) | 156 (68%) | 16 (7%) | 18 (8%) |

# 1.5 Do you think that the prescription behaviour of doctors will have an influence on the antibiotic resistance situation in your region?

|  | Not true | Rather not true | Rather true | True |
| --- | --- | --- | --- | --- |
| Total | 2 (1%) | 10 (3%) | 155 (44%) | 188 (53%) |
| Charité, Berlin | 1 (1%) | 4 (5%) | 39 (47%) | 39 (47%) |
| Uni. Würzburg | 1 (0%) | 6 (2%) | 116 (42%) | 149 (55%) |
| Early semesters | 2 (2%) | 4 (3%) | 65 (51%) | 57 (45%) |
| Advanced semesters | 0 (0%) | 6 (3%) | 90 (39%) | 131 (57%) |

Charité, Berlin / Uni. Würzburg: Fisher's exact test 0.2446

Advanced semester / early semester: Fisher's exact test 0.0276

# 1.6 Do you think that your future prescription behaviour as a doctor will have an influence on the antibiotic resistance situation in your region?

|  | Not true | Rather not true | Rather true | True |
| --- | --- | --- | --- | --- |
| Total | 1 (0%) | 29 (8%) | 154 (43%) | 171 (48%) |
| Charité, Berlin | 0 (0%) | 9 (11%) | 34 (41%) | 39 (47%) |
| Uni. Würzburg | 1 (0%) | 20 (7%) | 120 (44%) | 132 (48%) |
| Early semesters | 0 (0%) | 12 (9%) | 68 (54%) | 47 (37%) |
| Advanced semesters | 1 (0%) | 17 (7%) | 86 (38%) | 124 (54%) |

Charité, Berlin / Uni. Würzburg: Fisher's exact test 0.6567

Advanced semester / early semester: Fisher's exact test 0.0075

1. **Topic 2: Barriers**

# 2.1 In which areas do you think action should be taken to curb the increase in antibiotic resistance? (Please tick the areas that seem most important to you, multiple answers possible)

|  | Agricultural application | Patient compliance | Prescriptions by veterinarians | Prescribing outpatient physicians | Hospital prescriptions | Consulting and pharmaceutical care | Hospital hygiene | Hygiene in livestock farming | private hygiene/food preparation |
| --- | --- | --- | --- | --- | --- | --- | --- | --- | --- |
| Total | 286 (14%) | 239 (12%) | 255 (13%) | 320 (16%) | 258 (13%) | 146 (7%) | 246 (12%) | 182 (9%) | 47 (2%) |
| Charité Berlin | 65 (15%) | 50 (12%) | 57 (13%) | 75 (18%) | 55 (13%) | 25 (6%) | 50 (12%) | 39 (9%) | 8 (2%) |
| Uni. Würzburg | 221 (14%) | 189 (12%) | 198 (13%) | 245 (16%) | 203 (13%) | 121 (8%) | 196 (13%) | 143 (9%) | 39 (3%) |
| Early semesters | 102 (15%) | 75 (11%) | 83 (12%) | 109 (16%) | 86 (13%) | 48 (7%) | 86 (13%) | 61 (9%) | 24 (4%) |
| Advanced semesters | 184 (14%) | 164 (13%) | 172 (13%) | 211 (16%) | 172  (13%) | 98 (8%) | 160 (12%) | 121 (9%) | 23 (2%) |

# 2.2 Do you discuss the topic of antibiotic resistance with patients with infections when you have contact with such patients as part of your studies/internships?

|  | Almost always | Usually | Partly-partly | Rarely | Never |
| --- | --- | --- | --- | --- | --- |
| Total | 14 (4%) | 37 (10%) | 98 (28%) | 111 (31%) | 87 (24%) |
| Charité, Berlin | 2 (2%) | 4 (5%) | 21 (25%) | 29 (31%) | 25 (30%) |
| Uni. Würzburg | 12 (4%) | 33 (12%) | 77 (28%) | 82 (30%) | 62 (33%) |
| Early semesters | 6 (5%) | 13 (11%) | 28 (23%) | 38 (31%) | 36 (30%) |
| Advanced semesters | 8 (4%) | 24 (11%) | 70 (31%) | 73 (32%) | 51 (23%) |

Charité, Berlin / Uni. Würzburg: Fisher's exact test 0.2058

Advanced semester / early semester: Fisher's exact test 0.4273

# 2.3 Have you been to a doctor yourself with an infection in the last year?

|  | Yes | No | I don’t know |
| --- | --- | --- | --- |
| Total | 106 (30%) | 241 (68%) | 9 (3%) |
| Charité, Berlin | 30 (31% [26-47]) | 51 (61% [50-72]) | 2 (2% [0-8]) |
| Uni. Würzburg | 76 (28% [23-34]) | 190 (70% [64-75]) | 7 (3% [1-5]) |
| Early semesters | 46 (36% [28-45]) | 78 (61% [52-69]) | 4 (3% [1-8]) |
| Advanced semesters | 60 (26% [21-33]) | 163 (71% [65-77]) | 5 (2% [1-5%]) |

# 2.3.1 If yes, did the doctor talk to you about the topic of antibiotic resistance?

|  | Yes | No |
| --- | --- | --- |
| Total | 11 (10%) | 95 (90%) |
| Charité, Berlin | 3 (10%) | 27 (90%) |
| Uni. Würzburg | 8 (11%) | 68 (90%) |
| Early semesters | 3 (7%) | 43 (94%) |
| Advanced semesters | 8 (13%) | 52 (87%) |

# 2.3.2 Were you prescribed an antibiotic for the infection

|  | Yes | No | „“ |
| --- | --- | --- | --- |
| Total | 59 (17%) | 46 (16%) | 1 (0%) |
| Charité, Berlin | 11 (47%) | 16 (53%) | 0 (0%) |
| Uni. Würzburg | 45 (60%) | 30 (40%) | 1 (0%) |
| Early semesters | 21 (46%) | 24 (52%) | 1 (2%) |
| Advanced semesters | 38 (63%) | 22 (37%) |  |

# 2.3.2.1 If yes,…..

|  | „“ | Yes, but I took it differently than prescribed. | Yes, but I did not take it at all. | Yes, and I took it as prescribed. |
| --- | --- | --- | --- | --- |
| Total | 1 (2%) | 1 (2%) | 3 (5%) | 54 (92%) |
| Charité, Berlin | 0 | 0 | 0 | 14 (100%) |
| Uni. Würzburg | 1 (2%) | 1 (2%) | 3 (7%) | 40 (89%) |
| Early semesters | 0 | 1 (5%) | 0 | 20 (95%) |
| Advanced semesters | 1 (3%) | 0 | 3 (8%) | 34 (90%) |

# 2.3.3.2 If no, ….

|  | I obtained antibiotics on my own. | I did not take an antibiotic. |
| --- | --- | --- |
| Total | 1 (2%) | 45 (98%) |
| Charité, Berlin | 1 (6%) | 15 (94%) |
| Uni. Würzburg |  | 30 (100%) |
| Early semesters | 1 (4%) | 23 (96%) |
| Advanced semesters |  | 22 (100%) |

# 2.4 Have you ever taken an antibiotic without a doctor's prescription?

|  | Yes | No |
| --- | --- | --- |
| Total | 36 (10%) | 320 (90%) |
| Charité, Berlin | 6 (7% [3-15%]) | 77 (93% [85-97%]) |
| Uni. Würzburg | 30(11% [8-15%]) | 243 (89% [85-92%]) |
| Early semesters | 9 (7%) | 119 (93%) |
| Advanced semesters | 27 (12%) | 201 (88%) |

# 2.4.1 If yes, …..

|  | I procured antibiotics independently. | I bought it abroad without prescription. | I took leftover tablets. |
| --- | --- | --- | --- |
| Total | 13 (30%) | 12 (28%) | 18 (42%) |
| Charité, Berlin | 2 (29%) | 3 (43%) | 2 (29%) |
| Uni. Würzburg | 11 (31%) | 9 (25%) | 16 (44%) |
| Early semesters | 2 (18%) | 3 (27%) | 6 (55%) |
| Advanced semesters | 11 (34%) | 9 (28%) | 12 (38%) |

1. **Topic 3: Questions about antibiotic therapy / practical questions**

# 3.1 The following antibiotic is considered the antibiotic of first choice for infections with *Staphylococcus aureus*:

|  |  | Clindamycin | **Flucloxacillin** | I don’t know | Rifampicin |
| --- | --- | --- | --- | --- | --- |
| Total | 1 (0%) | 33 (9%) | **203 (57%)** | 101 (28%) | 18 (5%) |
| Charité, Berlin |  | 8 (10%) | **26 (31%)** | 39 (47%) | 10 (12%) |
| Uni. Würzburg | 1 (0%) | 25 (9%) | **177 (65%)** | 62 (23%) | 8 (3%) |
| Early semesters | 1 (1%) | 14 (11%) | **32 (25%)** | 67 (52%) | 14 (11%) |
| Advanced semesters |  | 19 (8%) | **171 (75%)** | 34 (15%) | 4 (2%) |

Charité, Berlin / Uni. Würzburg: Fisher's exact test <0.0001

Advanced semester / early semester: Fisher's exact test <0.0001

# 3.2 Acute bronchitis that persists for more than one week is usually an indication of antibiotic therapy.

|  |  | True | **False** | I don’t know |
| --- | --- | --- | --- | --- |
| Total | 1 (0%) | 45 (13%) | **209 (59%)** | 101 (28%) |
| Charité, Berlin | 0 (0%) | 8 (10%) | **41 (49%)** | 34 (41%) |
| Uni. Würzburg | 1 (0%) | 37 (14%) | **168 (62%)** | 67 (25%) |
| Early semesters | 1 (1%) | 20 (16%) | **41 (32%)** | 66 (52%) |
| Advanced semesters | 0 | 25 (11%) | **168 (74%)** | 35 (15%) |

Charité, Berlin / Uni. Würzburg: Fisher's exact test p=0.0185

Advanced semester / early semester: Fisher's exact test p<0.0001

# 3.3 Acute pyelonephritis in a young patient without concomitant disease is a clear indication for antibiotic therapy.

|  |  | **True** | False | I don’t know |
| --- | --- | --- | --- | --- |
| Total | 2 (1%) | **201 (56%)** | 57 (16%) | 96 (27%) |
| Charité, Berlin | 0 (0%) | **37 (45%)** | 9 (11%) | 37 (45%) |
| Uni. Würzburg | 2 (1%) | **164 (60%)** | 48 (18%) | 59 (22%) |
| Early semesters | 2 (2%) | **39 (30%)** | 21 (16%) | 66 (52%) |
| Advanced semesters | 0 (0%) | **162 (71%)** | 36 (16%) | 30 (13%) |

Charité, Berlin / Uni. Würzburg: Fisher's exact test 0.0004

Advanced semester / early semester: Fisher's exact test <0.0001

# 3.4 Antibiotic therapy for more than seven days^1^ is not usually required in the treatment of community-acquired pneumonia when there is clinical response.

|  |  | **True** | False | I don’t know |
| --- | --- | --- | --- | --- |
| Total | 3 (1%) | **164 (46%)** | 70 (20%) | 119 (33%) |
| Charité, Berlin | 0 (0%) | **36 (43%)** | 9 (11%) | 38 (46%) |
| Uni. Würzburg | 3 (1%) | **128 (47%)** | 61 (22%) | 68 (30%) |
| Early semesters | 2 (2%) | **28 (22%)** | 22 (17%) | 76 (59%) |
| Advanced semesters | 1 (0%) | **136 (60%)** | 48 (21%) | 43 (19%) |

Charité, Berlin / Uni. Würzburg: Fisher's exact test p = 0.0015

Advanced semester / early semester: Fisher's exact test p < 0.0001

^1^Authors’ note: At the time of the survey, the national guidelines for the treatment of community-acquired pneumonia (CAP) in adults generally recommended a treatment duration of seven days in the case of clinical response to the antibiotic therapy. Since 2021, there has been an updated guideline that recommends a treatment duration of five days for CAP if a response to the antibiotic therapy has already started (reference 18 in the manuscript).

1. **Topic 4: Knowledge acquisition**

# 4.1 What sources do you use to learn about antibiotic therapy and the development of antimicrobial resistance? (Multiple answers possible)

|  | Amboss-Online tool | Exchange with fellow students | No time | No need | Subject-specific webistes | Webistes for laymen | Medical textbooks | Medical guidelines | University course |
| --- | --- | --- | --- | --- | --- | --- | --- | --- | --- |
| Total | 266 (22%) | 175 (15%) | 49 (4%) | 7 (1%) | 98 (8%) | 44 (4%) | 174 (15%) | 152 (13%) | 232 (19%) |
| Charité, Berlin | 62 (23%) | 36 (13%) | 11 (4%) | 3 (1%) | 23 (8%) | 17 (6%) | 34 (12%) | 36 (13%) | 52 (19%) |
| Uni. Würzburg | 204 (22%) | 139 (15%) | 38 (4%) | 4 (0%) | 75 (8%) | 27 (3%) | 140 (15%) | 116 (13%) | 180 (20%) |
| Early semesters | 73 (19%) | 52 (14%) | 17 (4%) | 5 (1%) | 33 (9%) | 25 (7%) | 61 (16%) | 38 (10%) | 80 (21%) |
| Advanced semesters | 193 (24%) | 123 (15%) | 32 (4%) | 2 (0%) | 65 (8%) | 19 (2%) | 113 (14%) | 114 (14%) | 152 (19%) |

# 4.2 What media formats do you use in your studies in courses at the university or for your own studies at home? (Multiple answers possible)

|  | Apps | Blogs | Websites | Podcasts | Visual online tutorials |
| --- | --- | --- | --- | --- | --- |
| Total | 174 (26%) | 12 (2%) | 321 (48%) | 39 (6%) | 121 (18%) |
| Charité, Berlin | 39 (23%) | 2 (1%) | 79 (47%) | 9 (5%) | 39 (23%) |
| Uni. Würzburg | 135 (27%) | 10 (2%) | 242 (49%) | 30 (6%) | 82 (16%) |
| Early semesters | 59 (25%) | 5 (2%) | 113 (48%) | 10 (4%) | 49 (21%) |
| Advanced semesters | 115 (27%) | 7 (2%) | 208 (48%) | 29 (7%) | 72 (17%) |

# 4.3 In my previous studies, I have been able to acquire sufficient knowledge on the topic of appropriate antibiotic use and antimicrobial resistance.

|  | Not true | Tends not to be true | Tends to be true | True | No answer |
| --- | --- | --- | --- | --- | --- |
| Total | 59 (17%) | 143 (40%) | 134 (38%) | 17 (5%) | 3 (1%) |
| Charité, Berlin | 23 (28%) | 29 (35%) | 26 (31%) | 4 (5%) | 1 (1%) |
| Uni. Würzburg | 36 (13%) | 114 (42%) | 108 (40%) | 13 (5%) | 2 (1%) |
| Early semesters | 44 (34%) | 50 (39%) | 24 (19%) | 8 (6%) | 2 (2%) |
| Advanced semesters | 15 (7%) | 93 (41%) | 110 (48%) | 9 (4%) | 1 (0%) |

Charité, Berlin / Uni. Würzburg: Fisher's exact test p =0.02362

Advanced semester / early semester: Fisher's exact test p <0.0001

# 4.4 The importance of the topics of rational antibiotic therapy and antimicrobial resistance for my future work is overstated in my studies.

|  | No answer | Tends not to be true | Tends to be true | Not true | True |
| --- | --- | --- | --- | --- | --- |
| Total | 3 (1%) | 144 (40%) | 7 (2%) | 198 (56%) | 4 (1%) |
| Charité, Berlin | 0 (0%) | 26 (31%) | 3 (4%) | 54 (65%) | 0 (0%) |
| Uni. Würzburg | 3 (1%) | 118 (43%) | 4 (1%) | 144 (53%) | 4 (1%) |
| Early semesters | 2 (2%) | 47 (37%) | 5 (4%) | 73 (57%) | 1 (1%) |
| Advanced semesters | 1 (0%) | 97 (43%) | 2 (1%) | 125 (55%) | 3 (1%) |

Charité, Berlin / Uni. Würzburg: Fisher's exact test p = 0.0751

Advanced semester / early semester: Fisher's exact test p = 0.1803

# 4.5 In my studies, I learned what measures I could take myself to reduce the development of antimicrobial resistance.

|  | No answer | Tends not to be true | Tends to be true | Not true | True |
| --- | --- | --- | --- | --- | --- |
| Total | 2 (1%) | 105 (29%) | 168 (47%) | 38 (11%) | 43 (12%) |
| Charité, Berlin | 0 (0%) | 35 (42%) | 29 (35%) | 10 (12%) | 9 (11%) |
| Uni. Würzburg | 2 (1%) | 70 (26%) | 139 (51%) | 28 (10%) | 34 (12%) |
| Early semesters | 2 (2%) | 46 (36%) | 40 (31%) | 30 (23%) | 10 (8%) |
| Advanced semesters | 0 (0%) | 59 (26%) | 128 (56%) | 8 (4%) | 33 (14%) |

Charité, Berlin / Uni. Würzburg: Fisher's exact test p = 0.0228

Advanced semester / early semester: Fisher's exact test p < 0.0001

# 4.6 Which of the following formats do you consider to be suitable for imparting knowledge during studies on the topics of appropriate antibiotic use and antimicrobial resistance (please select a maximum of 5 formats):

|  | Learning-apps | E-learning | Training games | MOOC | Podcasts | Bedside teaching | Seminars | Lectures at university | Specific websites |
| --- | --- | --- | --- | --- | --- | --- | --- | --- | --- |
| Total | 145 | 145 | 167 | 107 | 67 | 140 | 300 | 209 | 121 |
| Charité, Berlin | 32 (10%) | 25 (8%) | 47 (15%) | 20 (7%) | 9 (3%) | 31 (10%) | 78 (25%) | 36 (12%) | 28 (9%) |
| Uni. Würzburg | 113 (10%) | 120 (11%) | 120 (11%) | 87 (8%) | 58 (5%) | 109 (10%) | 222 (20%) | 173 (16%) | 93 (8%) |
| Early semesters | 41 (9%) | 39 (8%) | 50 (11%) | 42 (9%) | 16 (3%) | 58 (13%) | 101 (22%) | 75 (16%) | 39 (8%) |
| Advanced semesters | 104 (11%) | 106 (11%) | 117 (12%) | 65 (7%) | 51 (5%) | 82 (9%) | 199 (21%) | 134 (14%) | 82 (9%) |

# 4.7 What content do you expect from an elective course or MOOC on appropriate antibiotic use and antimicrobial resistance? (Multiple answers possible)

|  | Features of individual antibiotics | Details of Pharmacokinetics | Epidemiology of multidrug-resistant pathogens | Interactive case studies | Guideline-based therapy | Microbiological diagnostics | Principles of appropriate antibiotic therapy | Resistance mechanisms | Selection and transmission | Modes of action of antibiotics |
| --- | --- | --- | --- | --- | --- | --- | --- | --- | --- | --- |
| Total | 247 (13%) | 69 (4%) | 182 (9%) | 239 (12%) | 308 (16%) | 84 (4%) | 285 (15%) | 166 (9%) | 50 (3%) | 296 (15%) |
| Charité, Berlin | 58 (13%) | 22 (5%) | 44 (9%) | 61 (13%) | 71 (15%) | 20 (4%) | 61 (13%) | 45 (10%) | 7 (2%) | 75 (16%) |
| Uni. Würz-burg | 189 (13%) | 47 (3%) | 138 (9%) | 178 (12%) | 237 (16%) | 64 (4%) | 224 (15%) | 121 (8%) | 43 (3%) | 221 (15%) |
| Early semesters | 92 (13%) | 35 (5%) | 72 (10%) | 83 (12%) | 95 (13%) | 32 (5%) | 91 (13%) | 82 (12%) | 16 (2%) | 108 (15%) |
| Advanced semesters | 155 (13%) | 34 (3%) | 110 (9%) | 156 (13%) | 213 (17%) | 52 (4%) | 194 (16%) | 84 (7%) | 34 (3%) | 188 (15%) |

# 4.8 Which channels would be a good way for us to reach you with news on the topic? (Multiple answers possible)

|  | Apps/Push Messages | E-Mail lists | Electronic newsletters | Facebook | Instagram | Twitter | Websites |
| --- | --- | --- | --- | --- | --- | --- | --- |
| Total | 74 (11%) | 324 (47%) | 74 (11%) | 37 (5%) | 72 (10%) | 6 (1%) | 106 (15%) |
| Charité, Berlin | 14 (8%) | 78 (47%) | 20 (12%) | 9 (5%) | 17 (10%) | 2 (1%) | 26 (16%) |
| Uni. Würzburg | 60 (11%) | 246 (47%) | 54 (10%) | 28 (5%) | 55 (10%) | 4 (1%) | 80 (15%) |
| Early semesters | 22 (9%) | 119 (50%) | 23 (10%) | 15 (6%) | 28 (12%) | 3 (1%) | 27 (11%) |
| Advanced semesters | 52 (11%) | 205 (45%) | 51 (11%) | 22 (5%) | 44 (10%) | 3 (1%) | 79 (17%) |

1. **Topic 5: Demographic data**

**5.1 Overview of demographic data**

|  |  | **Total** | **Berlin** | **Würzburg** | **Early semesters** | **Advanced semesters** |
| --- | --- | --- | --- | --- | --- | --- |
|  |  | 356 | 83 | 273 | 128 | 228 |
|  | **Berlin** |  |  |  | 46 | 37 |
|  | **Würzburg** |  |  |  | 82 | 191 |
| **Semester** | Median [IQR] | 7 [5,10] | 5 [3,8] | 8 [5,10] | 3 [2,5] | 9 [7,11] |
| **Gender** | Diverse (%) | 3 (0.8) | 1 (1.2) | 2 (0.7) | 0 | 3 |
|  | Male (%) | 106 (29.8) | 19 (22.9) | 87 (31.9) | 33 | 73 |
|  | Female (%) | 244 (68.5) | 62 (74.7) | 182 (66.7) | 93 | 151 |
|  | Not specified (%) | 3 (0.8) | 1 (1.2) | 2 (0.7) | 2 | 1 |
|  |  |  |  |  |  |  |
|  |  |  |  |  |  |  |
| **Work experience** | Yes (%) | 99 (27.8) | 17 (20.5) | 82 (30.0) | 37 | 62 |
|  | No (%) | 248 (69.7) | 65 (78.3) | 183 (67.0) | 88 | 160 |
|  | Not specified (%) | 9 (2.5) | 1 (1.2) | 8 (2.9) | 3 | 6 |

|  |  | **Total** | **Berlin** | **Würzburg** | **Early semesters** | **Advanced semesters** |
| --- | --- | --- | --- | --- | --- | --- |
| Professional experience in the health sector | Yes | 99 (27.8) | 17 (20.5) | 82 (30.0) | 37 | 62 |
|  | No | 248 (69.7) | 65 (78.3) | 183 (67.0) | 88 | 160 |
|  | N/A | 9 (2.5) | 1 (1.2) | 8 (2.9) | 3 | 6 |
| **From those:** |  |  |  |  |  |  |
| Nursing | Yes | 36 (10.1) | 9 (10.8) | 27 ( 9.9) | 17 | 19 |
| Physiotherapy | Yes | 5 (1.4) | 2 (2.4) | 3 (1.1) | 0 | 5 |
| Rescue Assistance | Yes | 37 (12.1) | 2 (4.8) | 35 (14.2) | 10 | 27 |
| Medical-technical assistant | Yes | 4 (1.1) | 0 (0.0) | 4 (1.5) | 2 | 2 |
| Pharmaceutical-technical assistant | Yes | 1 (0.3) | 0 (0.0) | 1 (0.4) | 0 | 1 |
| Others | Yes | 13 (3.9) | 3 (3.6) | 10 (4.0) | 7 | 11 |

**5.2 Details of professional experience in the health sector**
